# Postpartum sacral stress fracture: a case report

**DOI:** 10.1186/s12884-016-0873-4

**Published:** 2016-04-30

**Authors:** Nadeen Hilal, Anwar H. Nassar

**Affiliations:** Department of Internal Medicine, American University of Beirut Medical Center, Beirut, Lebanon; Ain Wazein Hospital, Ain Wazein, Lebanon; Department of Obstetrics and Gynecology, American University of Beirut Medical Center, PO Box: 11-0236, Riad El Solh, Beirut, 1107 2020 Beirut Lebanon

**Keywords:** Stress fracture, Sacrum, Postpartum, Back pain, Pregnancy

## Abstract

**Background:**

Stress fractures are classified as insufficiency and fatigue fractures. Insufficiency fractures occur when normal stresses are placed on bone with decreased mineralization and elastic resistance; whereas fatigue fractures occur when abnormal forces are applied to normal bone.

**Case presentation:**

We report a case of postpartum bilateral sacral fracture in the absence of documented osteoporosis in a 30 year old Lebanese female, thus satisfying the classification of fatigue fractures. Clinical presentation was mainly low back pain, pelvic pain, and abnormal gait.

**Conclusions:**

This case stresses the importance of including sacral fractures in the differential diagnosis of patients presenting with similar symptoms during pregnancy or the postpartum period.

## Background

Stress fractures are classified as insufficiency and fatigue fractures. Insufficiency fractures occur when normal stresses are placed on bone with decreased mineralization and elastic resistance; whereas fatigue fractures occur when abnormal forces are applied to normal bone, such as in tibial stress fractures in long distance runners [1]. Stress fractures occur secondary to repeated cyclic loading that eventually exceeds elastic resistance of bone [1].

Sacral insufficiency fractures were first recognized by Lourie in 1982 as a “distinct clinical entity of spontaneous osteoporotic fracture of the sacrum” [2]. Since their identification, several patient populations were found to be at increased risk. These include: the elderly, patients with weak bones from radiation therapy, and those with dysplastic, neoplastic, congenital, metabolic, or endocrine diseases [3].

Few cases of postpartum sacral fractures have been reported in the literature. Some cases were classified as insufficiency fractures [4] while others as fatigue fractures [2, 5–10]. In some cases, the authors were not sure if they were dealing with insufficiency or fatigue fractures [11–15]. We report a case of postpartum bilateral sacral fracture in the absence of documented osteoporosis.

## Case presentation

A 30-year-old gravida 1, para 1 presented because of bilateral buttocks and low back pain that started 10 days postpartum. The pain was dull in nature, was more severe on the right side, and was progressively increasing in intensity resulting in a limping gait. Pain worsened with walking, bending, and minor activity and was partially relieved with rest. Patient self-prescribed analgesics and non-steroidal anti-inflammatory drugs (NSAIDs) which used to relief pain transiently.

The patient is otherwise healthy with no history of metabolic bone disease, menstrual irregularities, previous fractures, eating disorders, or strenuous athletic activity. No family history of osteoporosis was reported. The course of pregnancy was smooth with no remarkable back, hip, or buttocks pain reported. The total weight gained during pregnancy was 14 kg. She was maintained on supplemental iron, calcium (1000 mg daily), and vitamin D (800 international units (IU)) daily as of the second trimester. At term, she presented in labor and received epidural anesthesia. The first stage of labor was 7 h and second stage was 100 min. She had an uneventful, spontaneous, normal vaginal delivery without the need for forceps or vacuum application. The baby was healthy and weighed 3350 g.

Two weeks after onset of her symptoms, the patient sought medical attention because of persisting pain and abnormal gait. On physical exam, the patient was of average body habitus with height 164 cm and weight 60 kg. Mobility of the lumbosacral spine was within normal limits. Tenderness around the upper gluteal region and sacrum was noted bilaterally; more evident on the right side.

**Fig. 1:**
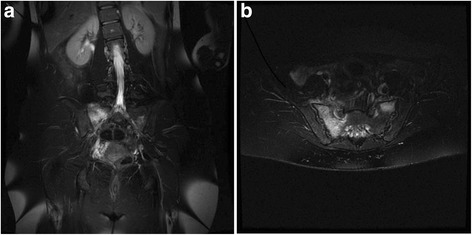
MRI of pelvis. **a** coronal T2 demonstrating bone marrow edema with non-displaced fracture line on right sacrum. **b** axial T2 demonstrating bone marrow edema in both sacral bones

Pelvic magnetic resonance imaging (MRI), performed to rule out soft tissue lesions, demonstrated moderate-to-severe bone marrow edema involving the right sacrum along its entire length with a non-displaced fracture line. Mild edema was also seen involving the left sacrum. Results of the MRI are displayed in Fig. 1a and b.

Dual-energy X-ray absorptiometry was performed to rule out pregnancy-associated osteoporosis. It revealed normal lumbar spine and left forearm mineral densities with low normal values at the femur bone (Table 1). Basic metabolic workup including complete blood count, thyroid stimulating hormone, parathyroid hormone, vitamin D, serum calcium, and urinary calcium levels were normal.

**Table 1:** Results of Dual-energy X-ray Absorptiometry

Skeletal region	Bone density at diagnosis of fracture (g/cm^2^)	Bone density at follow up after 5 months (g/cm^2^)	SD^a^ above or below the mean for young adults at diagnosis of fracture (Z score)	SD^a^ above or below the mean for young adults at follow up after 5 months (Z score)
Spine (L1-L4)	0.875	0.878	−1.6	−1.5
Femur: Total				
Neck	0.706	0.733	−1.9	−1.7
	0.642	0.684	−1.9	−1.5
Forearm	0.638	NA^b^	−0.9	NA^b^

^a^ *SD* standard deviation, ^b^ *NA* not available

Patient was treated with relative rest, analgesics, and NSAIDs. Empirically, and based on no solid evidence, she was also given oral vitamin D (50,000 IU per week) and calcium (1 g daily) supplementation to facilitate and fasten bone healing. Breastfeeding was not interrupted. Vitamin D was continued for a total of 4 months. One month later, patient symptoms started to improve gradually requiring less pain medications. Pain completely resolved and gait was restored around 6 months postpartum. No follow up MRI was performed and the patient was considered to have a healed fracture based on resolution of her symptoms.

To note, after 5 months of the diagnosis of sacral fractures, the patient underwent a follow up dual-energy X-ray absorptiometry. This study was performed as a follow up after improvement of symptoms and as a baseline before a planned second pregnancy. The results are outlined in Table 1.

### Discussion

This article presents a case of postpartum spontaneous sacral fracture in the absence of documented osteoporosis. Risk factors for sacral stress fractures during pregnancy or in the first weeks postpartum include vaginal delivery of large for gestational age infants, increased lumbar lordosis, excessive weight gain and a rapid vaginal delivery (precipitous labor) [16]. Other risk factors include vitamin D deficiency, anticoagulant therapy with heparin and transient osteoporosis associated with pregnancy and lactation. Our patient did not suffer from any of those risk factors. Since all the workup done including dual-energy X-ray absorptiometry was normal; the fracture can be classified as a sacral fatigue fracture. However, there is a high probability that we might have been dealing with a case of pregnancy-associated osteoporosis due to two reasons. The first one is the result of the first dual-energy X-ray absorptiometry that was performed 5 weeks postpartum. The International Society for Clinical Densitometry recommends the use of BMD Z-scores in premenopausal women, where a Z-score of lower than −2.0 should be interpreted as “below the expected range for age” [17]. Thus, despite the fact that the results do not fulfill criteria of low bone density (Z score < −2) in premenopausal women, it is evident that the bone density is borderline low, especially at the femur where Z score was - 1.9. Thus, there might be a possibility that these numbers were lower during pregnancy and the immediate postpartum period and have already started to correct by the time the study was done. Another reason is that the follow up dual-energy X-ray absorptiometry that was performed 5 months later revealed an increase in the bone density at the spine and femur. Densitometry measurement of the forearm was not repeated. Another reason why this case can be regarded as a complication of pregnancy-associated osteoporosis is the timing of the event, which occurred in the immediate postpartum period; the affected skeletal region being the spine; and the lack of other reasons or risk factors for fractures. However, the possibility of pregnancy-associated osteoporosis remains a hypothesis that cannot be fully proven in this case.

## Conclusion

Since their initial description, little knowledge has been added concerning the pathogenesis, patient population, clinical manifestations, diagnostic techniques, and treatment options of sacral stress fractures. Thus, they have remained an unexplored clinical entity and are likely a frequently underdiagnosed cause of low back or pelvic pain. This case and similar cases previously reported suggest that sacral stress fractures should be considered in the differential diagnosis of low-back pain, buttock pain, and abnormal gait during pregnancy and the postpartum period. Delays in the diagnosis might be very painful for the women affected.

### Ethics approval and consent to participate

Not applicable.

### Consent for publication

Written informed consent was obtained from the patient for publication of this case report and any accompanying images. A copy of the written consent is available for review by the Editor of this journal.

### Availability of data and materials

Not applicable.

## Abbreviations

IU: international units
MRI: magnetic resonance imaging
NSAIDS: non-steroidal anti-inflammatory drugs
